# Prevalence of bacterial vaginosis and associated risk factors in pregnant women receiving antenatal care at the Kumba Health District (KHD), Cameroon

**DOI:** 10.1186/s12884-019-2312-9

**Published:** 2019-05-10

**Authors:** Yiewou Marguerithe Kamga, John Palle Ngunde, Jane-Francis K. T. Akoachere

**Affiliations:** 1 Department of Microbiology and Parasitology, Faculty of Science, University of Buea, Buea, Cameroon; 2 Department of Nursing, Faculty of Health Sciences, University of Buea, Buea, Cameroon

**Keywords:** Bacterial vaginosis, Risk factors, Pregnant women, Cameroon

## Abstract

**Background:**

Bacterial vaginosis (BV) is a common reproductive tract disorder in women of child bearing age, accounting for one third of vaginal infections. It is characterized by an increase in vaginal pH, decreased Lactobacilli, and overgrowth of facultative and anaerobic bacteria. Studies have consistently shown BV to be a risk factor for adverse obstetric and gynecological outcomes. BV is believed to play a critical role in the transmission of sexually transmitted infections. Its aetiology and risk factors are poorly understood. This study determined the prevalence and risk factors for BV among pregnant women in Kumba Health District (KHD) Cameroon to generate findings that could guide the design of interventions for prevention of infection and associated poor pregnancy outcomes.

**Methods:**

A structured questionnaire was administered to 309 women seeking antenatal care (ANC) in three health facilities in KHD between May to July 2016, to capture data on demographic, gynecological and obstetric characteristics, and hygiene behavior. High vaginal swabs (HVS) collected from these women were gram stained, examined under a microscope and BV evaluated by Nugent scoring. Chi square (χ^2^) test was used to determine the relationship between BV and factors investigated. Statistical significance was set at *p* < 0.05.

**Results:**

The prevalence of BV was 26.2%. Nine point 1 % of participants had a mixed infection with *Candida*. BV was higher (29.5%) in participants from the rural area (χ^2^ = 8.609. *P* = 0.014), those who did not use antibiotics (31.9%) prior to the study (χ^2^ = 12.893, *P* = 0.002) and women with no history of a genital tract infection (χ^2^ = 18.154, *P* = 0.001). There was a significant difference in prevalence with respect to gestation age (χ^2^ = 13.959, *P* = 0.007) with the highest occurring in women in the second trimester (31.7%). Women who practiced douching (χ^2^ = 23.935, *P* = 0.000) and those who did not wash pants with disinfectant (χ^2^ = 7.253, *P* = 0.027) had a high prevalence.

**Conclusion:**

BV could be a health concern among pregnant women in study area. BV prevalence was affected by some hygiene behaviors, socio-demographic and clinical factors. Screening and treatment of positive cases during antenatal visits to prevent adverse outcomes, as well as education of women on vaginal hygiene is highly recommended.

## Background

Bacterial vaginosis (BV) is an extremely common reproductive tract disorder worldwide in women of child bearing age, accounting for one third of vaginal infections [1]. It is characterized by malodorous vaginal discharge with a fishy odour upon addition of 10% potassium hydroxide (KOH), and the presence of clue cells, few or no lactobacilli and a few or no polymorphonuclear leucocytes. Bacteria vaginosis can occur in pregnant and non-pregnant women. Some cases may present only with vaginal discharge and no inflammation. The prevalence is in general, higher in parts of Africa [2] but in other parts of the world, it has been shown to vary with race and ethnic group [3].

Bacteria vaginosis is a polymicrobial syndrome in which there is a change in the complex balance of microflora in the vagina. There is a decrease in Lactobacilli and an overgrowth of facultative and anaerobic bacteria either singly or in combination. These bacteria include *Gardnerella vaginalis, Bacteroides fragilis, Atopobium vaginae, Prevotella, Peptostreptococcus, Mobiluncus, Sneathia, Leptotrichia, Propionibacterium, Fusobacterium, Veillonella spp, Mycoplasma hominis, Ureaplasma urealyticum*, *Streptococcus* spp., *Staphylococcus* spp., *Bifidobacterium* spp., *Fusobacterium* spp., and members of the Enterobacteriaceae*.* This list continues to expand. These organisms are usually present in small numbers in a healthy vagina. *Mobiluncus* species are rarely found and thus their presence is a marker for diagnosis of BV. The specific causes of BV are poorly understood. A recent study [4] has reported a significant higher percentage of healthy women colonized by *G. vaginalis* indicating that colonization of the vagina by *G. vaginalis* does not always lead to BV development. Hyman et al. [5] investigating the vaginal epithelium flora of healthy women reported the population of Lactobacilli to range from 0 to 100%, with *Bifidobacterium, Gardnerella, Prevotella, Pseudomonas* or *Streptococcus* predominating in vagina where *Lactobacillus* was absent.

The associated risk factors of BV are also poorly understood as reports on risk factors have been conflicting. There are reports on the association of BV with demographic, behavioral and clinical characteristics of pregnant women [6–10] while other studies report no association with some of these factors [11]. Also, there has been contradicting reports on sexual transmission as a necessary prerequisite to the acquisition of BV. Some studies have reported sexual involvement while others detected BV in sexually inexperienced women [12]. As a result, Verstraelen [12] suggested BV to be considered a sexually enhanced disease (SED) rather than a sexually transmitted disease (STD). Other studies have reported its association with multiple sex partners [13]. Because prevention strategies of any disease target risk factors, knowledge of risk factors of BV is important in the design of effective interventions to prevent poor pregnancy outcomes, particularly as many cases are asymptomatic [14].

Bacteria vaginosis is a common problem occurring among pregnant women and studies have consistently shown BV to be a risk factor for adverse obstetric and gynecological outcomes such as pre-term labor and delivery [8, 15, 16], premature rupture of membranes, low birth weight [17], spontaneous abortion [18], postpartum infections such as endometritis [19] and caesarean section wound infections [20]. Since most cases remain asymptomatic early detection, especially among pregnant women is essential for timely treatment and prevention of related complications and poor pregnancy outcomes.

Bacteria vaginosis has emerged as a public health problem due to its association with sexually transmitted infections (STIs) [1, 21, 22]. Several studies have demonstrated that it can play a critical role in transmission, as a disturbed vaginal ecology by BV makes the vaginal environment more permissive for acquisition of infection and hence impact disease dynamics of STIs [3, 23, 24]. The acidic environment, hydrogen peroxide and bacteriocins produced by lactobacilli in a healthy vagina make the environment unfavorable for the proliferation of bacteria vaginosis associated bacteria and sexually transmitted pathogens [1]. The modification of the vaginal microbiome in bacteria vaginosis enhances susceptibility to STIs. Therefore timely detection and therapeutic intervention and eradication could be an important plan for primary prevention of STIs.

Studies on BV in pregnancy have been conducted in several countries [8, 11, 14], but there is insufficient data from Cameroon, with regards to prevalence and associated risk factors among pregnant women. Studies on BV in Cameroon so far involved the epidemiology of *T. vaginalis*, *G. vaginalis* and *Candida albicans* co-infection [25], prevalence and risk factors on sexually active women [6] as well as women with STIs in Dschang seeking gynecological care [26]. None of these studies focused on pregnant women. However, Mbu et al. [27] included BV in their study comparing the prevalence of gynecological conditions among HIV infected and non-infected pregnant women receiving antenatal care. In resource poor countries including Cameroon, pregnant women on antenatal care are often screened for sexually transmitted infections including syphilis and HIV but rarely other treatable STIs or BV. In some instances, pregnant women with vaginal discharge are treated for vaginal candidiasis without laboratory investigations. To avoid pregnancy complications and poor outcomes due to BV, knowledge of its prevalence and associated risk factors is important for treatment and prevention interventions. This study was conducted to determine the prevalence of bacterial vaginosis and associated risk factors among pregnant women receiving antenatal care at the Kumba Health District (KHD) Cameroon.

## Methods

### Study area and design

This study was conducted in Kumba Health District (KHD). Kumba Health District spans through Kumba 1, 2 and 3 Sub-divisions, and parts of Mbonge Sub-division. It is situated at 4.64° North latitude, 9.45° East longitude, and is 258 m above sea level. Kumba is one of the major towns in Cameroon, and has about 144,413 inhabitants. Kumba Health District is the largest of the eighteen health districts of the South-West Region of Cameroon. It is home to 20% (265, 071) of the population of South-West Region. Its health needs are satisfied by 30 health facilities: 5 general hospitals and 25 health centers, which are located in 12 health areas, 5 of which are situated within Kumba township.

This descriptive cross-sectional study was conducted in 3 major health care facilities: District Hospital Kumba (DHK), Kumba Urban Sub-Divisional Medical Center (CMA Kumba Urban), Sub- Divisional Medical Center Ntam III (CMA Ntam).

### Study participants

Study participants comprised pregnant women visiting the obstetric and gynecological units of participating health facilities for ANC and who gave a written consent/assent. Women who were bleeding, had inserted some drugs in the vagina and those for whom consent was not received were excluded from this study.

### Sample size

The sample size for the study was 309 women, calculated using the formula by Daniel [28] based on a prevalence of BV of 15.2% reported in HIV negative pregnant women in Yaoundé, Cameroon [27]. Margin of error was 5% (0.05).

### Sampling technique and data collection

The convenient sampling was used because the time for the collection of data was short. Data was collected from May to July 2016 using a pre-tested structured questionnaire that was designed to capture data on participant’s demographic, gynecological and obstetric characteristics and hygiene behaviour. Demographic data included age, level of education, marital status, place of residence, while gynecological data collected comprised gestational age, history of sexual transmitted infection or genital tract infection; obstetric data included gravidity, parity, previous pregnancy outcomes while data on hygiene behaviour included care for pants and douching. After physical and obstetrical examination, a high vaginal swab (HVS) was collected in the laboratory by instructing the participant to assume a lithotomy position and a sterile unlubricated speculum inserted into the vagina. Two sterile cotton swabs were used to swab the vagina walls and the samples properly labeled. One was streaked on a slide containing normal saline and 10% KOH (wet mount) and the other was streaked on a clean grease-free slide, air-dried, heat fixed, Gram stained, and examined under a microscope using a X40, X100 objectives. The wet mount was observed under X10 and X40 objectives to identify *Candida* and *Trichomonas vaginalis* (TV).

### Ethical considerations

Ethical clearance was obtained from the Institutional Review Board of the Faculty of Health Sciences, University of Buea, Cameroon. Administrative authorization was obtained from the Delegation of Public Health for the South West Region. The Directors of participating hospitals granted approval for the study to be conducted in their facility.

Participation in the study was voluntary and a written informed consent/assent was obtained from the participants after the objectives of the study were explained to them. For participants less than 21 years, consent was obtained from their parents/guardians. For those who could not read and write, the questionnaire was administered to them in pidgin English, a local type of English and in place of signature, their thumb print was taken. Confidentiality was ensured by assigning codes to questionnaires and samples. Sample collection was done in a less invasive manner. All participants received their results after diagnosis and those positive for bacterial vaginosis were notified, and treatment prescribed by attending physician or obstetrician.

### Data analysis

The Nugent scoring [29] was used to evaluate the presence of BV. Two research assistants who are laboratory technologists had a refresher training on Nugent scoring prior to the study. This was to ensure good and uniform practice in preparation and reading of gram stains. The Nugent is a standardized 0–10 point scoring system for evaluation of Gram stained vaginal smears. It is score is calculated by assessing for the presence of large Gram-positive rods (*Lactobacillus* morphotypes; decrease in *Lactobacillus* scored as 0 to 4), small Gram-variable rods (*Gardnerella vaginalis* morphotypes; scored as 0 to 4), and curved Gram-variable rods (*Mobiluncus* spp. morphotypes; scored as 0 to 2) (Table 1). A score of 0–10 is generated from combining three other scores. A score of 0–3 is considered negative for BV, 4–6 is considered intermediate and 7+ is considered indicative of BV. At least 10–20 high power (1000× oil immersion) fields are counted and an average determined.

**Table 1:** A Scoring system (0 to 10) for Gram-stained vaginal smears

*Lactobacillus* morphotypes	*Gardnerella/Bacteroides* morphotypes	Curved Gram variable rods
Score of 0 for > 30	Score of 0 for 0	Score of 0 for 0
Score of 1 for 15–30	Score of 1 for < 1 (this is an average, so results can be > 0, yet < 1)	Score of 1 for < 5
Score of 2 for > = 1–14	Score of 2 for 1–4	Score of 2 for > = 5
Score of 3 for < 1 (this is an average, so results can be > 0, yet < 1)	Score of 3 for 5–30	
Score of 4 for 0	Score of 4 for > 30	

Data was entered into Epi info 7 spread sheets and then exported into SPSS version 20 for windows (SPSS Inc., Chicago USA). Descriptive information was expressed as percentages. The Chi-square (χ^2^) test was used to test the relationship between BV and factors investigated. Statistical significance was set at *p* < 0.05.

## Results

### Characteristics of study participants

Three hundred and nine (309) women were enrolled in this study comprising, 83 (26.9%), 98 (31.7%) and 128 (41.4%) from DHK, CMA Ntam III and CMA Kumba Urban respectively. The age range was 18 to 42 years with mean age 26.97 years. The highest number of participants was in the age group of 23 to 27 years (33.3%) (Table 2). With regards to place of residence, over half (63.4%) of the study participants resided in the urban area. Most (72.5%) participants were married and had secondary education (36.9%). Two hundred and twenty eight (73.8%) women were multigravida with a mean gravidity of 2.86. Two hundred and seven (66.9%) of them had delivered and the mean parity was 1.48. All participants had single sexual partners (Table 2).

**Table 2:** Characteristics of study participants

Characteristics	Number enrolled	Percentage %
Study sites		
DHK	83	26.9
CMA Ntam III	98	31.7
CMA Kumba Urban	128	41.4
TOTAL	309	
Marital status		
Single	84	27.2
Married	224	72.5
Widow	1	0.3
Age group (years)		
18–22	72	23.3
23–27	103	33.3
28–32	91	29.5
33–37	33	10.7
38–42	10	3.2
Educational Level		
No education	6	1.9
Primary	99	32
Secondary	114	36.9
Tertiary	90	29.1
Residence		
Urban	196	63.4
Rural	113	36.6
Number of sexual partners		
1	309	100
>1	0	0
Parity		
0	102	33.0
1–4	194	62.8
> 4	13	4.2

### Prevalence of BV and co-infections

Bacterial vaginosis was detected in 81 (26.2%) women. Among the BV associated organisms detected, *Gardnerella vaginalis* was the commonest morphotype accounting for approximately 55.0%, followed by *Bacteroides fragilis* (26.5%) while *Mobiluncus* (11.3%) was the least. Other infections detected were *Candida* (27.8%, 86/309) and *Trichomonas vaginalis* (1%, 3/309). Mixed infection of *Candida* and BV was detected in 28 (9.1%) women but none with women with *Trichomonas vaginalis* (Table 3).

**Table 3:** Prevalence of BV and co-infections

Type of infection	Number positive	% positive
Bacteria vaginosis	81	26.2
Candida	86	27.8
Candida/BV	28	9.1
Trichomonas vaginalis	3	1
T.vaginalis/BV	0	0

### Occurrence of BV and relationship3with socio-demographic characteristics of participants

With respect to study site, the highest prevalence was among participants from CMA Ntam III (32.8%). The difference was significant (χ^2^ = 43.1, *P* = 0.001). The prevalence of BV decreased with increasing age from 18 years to 42 years. Most women with BV were in the age group 18–22 (29.2%) years (Table 4). There was no significant difference with respect to age (χ^2^ = 12.196, *P* = 0.143). Bacterial vaginosis was higher in married women (26.8%) than single women (25.0%) but the difference was not significant (χ^2^ = 5.883, *P* = 0.208). Bacterial vaginosis was significantly higher in women from rural parts of the health district (29.5%) than those from urban area (24.5%) (χ^2^ = 8.609. *P* = 0.014) and decreased with increase in level of education, being highest among participants who had no education (33.3%) but the difference was not significant (χ^2^ = 2.373, *P* = 0.882).

**Table 4:** Relationship between socio-demographic characteristics and BV prevalence

Characteristics	Number enrolled	Number positive for BV n (%)	χ^2^, *P*-value
Study site			
DHK	83	25 (30.1)	43.1, < 0.001
CMA Ntam III	98	42 (32.8)	
CMA Kumba Urban	128	14 (14.3)	
TOTAL	309	81 (26.2)	
Marital status			
Single	84	21(25.0)	5.883, 0.208
Married	224	60 (26.8)	
Widow	1	0 (0.0)	
Age group (years)			
18–22	72	21 (29.2)	12.196, 0.143
23–27	103	26 (25.2)	
28–32	91	24 (26.4)	
33–37	33	10 (23.3)	
38–42	10		
Educational Level			
No education	6	2 (33.3)	2.373, 0.882
Primary	99	27 (27.3)	
Secondary	114	32 (28.1)	
Tertiary	90	21 (23.3)	
Residence			
Urban	196	48 (24.5)	8.609, 0.014
Rural	113	33 (29.5)	

Key: *DHK* District Hospital Kumba, *CMA* Centre Medicaled’Arrondissement

### Relationship between bacterial vaginosis, hygiene behavior, and obstetric and gynecological history of participants

Bacterial vaginosis was significantly higher in participants who did not use antibiotics prior to sample collection (31.9%) compared to those who did (15.2%) (χ^2^ = 12.893, *P* = 0.002). The prevalence was significantly higher in participants with no history of a genital tract infection (26.9%) than women who reported a previous infection (χ^2^ = 18.154, *P* = 0.001). Participants in the second trimester (31.7%) had the highest prevalence while those in the third trimester (18.0%) had the lowest. There was a significant difference in prevalence with respect to gestation age (χ^2^ = 13.959, *P* = 0.007) (Table 5). Although the other factors investigated had no significant influence, the prevalence was highest in women with parity greater than 4 (38.5%), and in women who reported perinatal death (25.7%). The prevalence was higher in HIV negative women (26.3%) compared to those HIV infected (21.4%) and also in women who were primigravida (30.9%) than in multigravida (24.6%).

**Table 5:** Relationship between Bacterial vaginosis and obstetric and gynecological

Clinical Characteristic	Number positive for BV	Percentage positive	Statistics χ^2^ (*P*-value)
Use of antibiotics			
Yes (*n* = 105)	16	15.2	12.893 (0.002)
No (*n* = 204)	65	31.9	
HIV Status			
Positive (*n* = 14)	3	21.4	0.822 (0.935)
Negative (*n* = 293)	77	26.3	
Genital tract infection			
Yes (*n* = 124)	32	25.8	18.154 (0.001)
No (*n* = 182)	49	26.9	
Gestational age			
1st Trimester (n = 29)	6	20.7)	13.959 (0.007)
2nd Trimester (*n* = 180)	57	31.7	
3rd Trimester (*n* = 100)	18	18.0	
Previous pregnancy loss			
Perinatal (*n* = 35)	9	25.7	9.569 (0.144)
Spontantaneousab (*n* = 38)	7	18.4	
Induced ab (*n* = 15)	3	20.0	
Gravidity			
Primigravida	81	25 (30.9)	3.338, 0.188
Multigravida	228	56 (24.6)	
Parity			
0	102	30 (29.4)	6.340, 0.175
1–4	194	46 (23.7)	
> 4	13	5 (38.5)	

ab: abortion

With regards to the behavioural characteristics of participants, the prevalence of bacterial vaginosis was significantly higher in women who practiced douching (χ^2^ = 23.935, *P* = 0.000) and those who did not wash their under pants with disinfectant (χ^2^ = 7.253, *P* = 0.027) compared to those who did not douch or disinfect their pants (Table 6). The prevalence was higher in women who used soap for douching (27%), did not dry their pants under sunlight (31.2%), did not iron pants (27.3%), did not share toilet facility (27.9%) and wore wet pants (26.3%) but the differences were not significant.

**Table 6:** Relationship between bacterial vaginosis and behavioural characteristics

Vaginal hygiene		Number enrolled	Number positive for BV (%)	χ^2^ (*P*-value)
Vaginal douching	Yes	221	74 (33.5)	23.935 (0.000)
	No	87	7 (8.0)	
Usage of soap in douching	Yes	126	34 (27.0)	4.631 (0.327)
	No	182	46(25.3)	
Dry pants under sunlight	Yes	138	28 (20.3)	8.504 (0.075)
	No	170	53 (31.2)	
Wash pants with disinfectant	Yes	137	27 (19.7)	7.253 (0.027)
	No	172	54 (31.4)	
Iron pants	Yes	23	3 (13.0)	4.543 (0.103)
	No	286	78 (27.3)	
Share toilet facility	Yes	130	31 (23.8)	1.372 (0.504)
	No	179	50 (27.9)	
Wear wet pant	Yes	19	4 (21.1)	3.140 (0.535)
	No	289	76 (26.3)	

## Discussion

The overall prevalence of BV in this study was 26.2%, higher than 15.2% reported in HIV negative pregnant women by Mbu et al. [27] in Yaounde, Cameroon. Our study employed the Nugent scoring for diagnosis of BV whereas Mbu et al. [27] used the Amsel criteria [30] which has been reported to be inferior to the Nugent scoring [31]. Nsagha et al. [25] reported a similar prevalence in Yaounde, Cameroon but their study was on non-pregnant women. Our prevalence is higher than 24% reported by Kesah et al. [26]. Findings of present study together with previous reports show that BV could be serious reproductive health concern among women in Cameroon. Compared to similar studies in other African countries our prevalence was similar to 26% in Lagos, Nigeria [8], higher than 19.4% in Ethiopia [14] and 17.6% in South Africa [32] but lower than 28.5% in Tanzania [11], 49.8% in Khartoum-Sudan [33] and 60% in Nigeria [34]. Differences in prevalence reported in different settings could be due to environmental, behavioral, socioeconomic status and stressor differences with geographical variation [35]. The high prevalence of BV reported in present study may be due to the high rate of vaginal douching (221/309, 71.5%) observed among study participants. Women who practiced douching had a significantly higher prevalence of BV (χ^2^ = 23.935, *P* = 0.001) than those who were not douching similar to reports from elsewhere [36]. Previous studies in Cameroon have also reported douching as a common practice among women [6, 26], as they believe that thorough bathing must involve douching. This is appalling considering the high level of education of participants: 36.9% secondary, 29.1% higher education. Other behavioural practices investigated such as use of soap for douching, drying of pants under sunlight, washing of pants with disinfectant, ironing of pants, wearing of wet pants and sharing of toilet facilities did not have any significant effect on the prevalence of BV. Chemicals in soap out may adversely affect harmless friendly bacteria in the genitalia thus favoring the proliferation of BV pathogens. *Gardnerella* was the commonest morphotype. Mixed infections of BV and *Candida* were detected in 9.1% of participants. *T. vaginalis* was detected in 1% of women as a monoinfection. These findings contradict the study of Sobel et al. [37] which reported BV co-infections with *T. vaginalis* to be more common than with other pathogens.

The highest prevalence of BV was recorded in women aged 18–22 years similar to Ibrahim et al. [38]. However, Mengistie et al. [14] recorded highest prevalence in women aged 21–29 years. Individuals in these age groups are most active sexually and thus at the highest risk of BV and STDs [38, 39]. Contrary to our findings, Ranjit et al. [36] reported the highest prevalence of BV in women 30–40 years but these were non-pregnant women. Primigravidae participants had a higher rate of BV compared to multigravidae, though the difference was not significant, corroborating the findings of a similar study in Nigeria [34]. Some multigravidae participants have been exposed to antenatal care where they received health education on vaginal hygiene practices and good health behaviors while the primigravidae were still in-experienced. However, Ibrahim et al. [38] reported the highest prevalence in the multigravida, contradicting our finding. The prevalence of BV was highest in the second trimester of pregnancy, similar to the report of Ibrahim et al. [38] but contradicts the findings of Awoniyi et al. [34] whose prevalence decreased with increase in trimester. More than half of our study participants (180/309, 58.3%) were in the second trimester and this might have influenced our results. However, there was no significant difference in prevalence of BV with regards to gestation age. Since BV has been associated with second trimester miscarriages [18], the highest prevalence in women in the second trimester in present study is a concern.

There was no significant difference in the prevalence of bacterial vaginosis between HIV positive and HIV negative women. This corroborates the findings of Shayo et al. [11] who reported no association between BV and HIV infection but contradicts previous studies [23, 40, 41] that have highlighted a strong association of both symptomatic and asymptomatic bacterial vaginosis and HIV infection. BV was higher in HIV negative women (26.3%) compared to those infected with HIV (21.4%) contrary to Mbu et al. [27] who reported a higher prevalence in HIV infected pregnant women (21.2%) than in HIV negative women (15.2%). The lower prevalence in HIV positive women observed in our study could be as a result of the improvement in maternal health care as a consequence of the utilization of HIV treatment programmes which might not have been the case during the study of Mbu et al*.* [27].

There was no significant difference in prevalence of bacterial vaginosis with respect to marital status, similar to Shayo et al. [11], although the prevalence was higher in married women than unmarried women. Ranjit et al. [36] reported a higher prevalence in unmarried women, contradicting our findings. The majority of participants in the present study were married (224/309, 72.5%) and this may account for this observation. There was no significant difference in BV prevalence with respect to educational level though the highest prevalence observed in women who had no education. There was a significant difference in prevalence of BV with regards to place of residence of participants. Women residing in the rural parts of KHD had a higher prevalence (29. 5%) than those in the urban area (24.5%) (χ^2^ = 8.609, *P* = 0.014). The higher prevalence of BV in rural areas may be linked to poverty. Although this study did not investigate income level as a risk factor, Cameroon’s poverty is a rural phenomenon [42]. Previous studies have associated low income status to a higher prevalence of bacterial vaginosis [14, 36]. Poverty affects good hygiene practices and individual hygiene has been reported to influence prevalence of BV [7]. This may explain our findings.

Participants who recently used antibiotics prior to this study had a significant lower prevalence of BV than those who did not use antibiotics (χ^2^ = 12.893, 0.002). Likewise, those who reported a previous genital tract infection had a significant lower prevalence of BV than those who did not (χ^2^ = 18.154, 0.001). Bacteria vaginosis is treated using antibiotics. Antibiotics used for BV or for other infections may have a protective or curative effect on BV reducing its occurrence in such individuals.

The major strength of this study is the rigour in arriving at the diagnosis of bacterial vaginosis. However the limitations of this study need to be highlighted. This study focused on pregnant women receiving antenatal care as such did not give the prevalence of Bacterial vaginosis in the general population. Also, only women who sought antenatal care in the participating hospitals within the period of our study were recruited, thus, are not a representation of the annual population served by these hospitals. In addition the results will not apply to other women seen in other hospitals in study area at the same period.

## Conclusion

The high prevalence of BV obtained shows it could be health concern among pregnant women receiving antenatal care at the Kumba Health District. Bacterial vaginosis prevalence was affected by some hygiene behaviours, socio-demographic and clinical characteristics. We therefore recommend that pregnant women seeking antenatal care in study area should be routinely screened for BV and other genital tract infections apart from those under routine investigation, and positive cases treated to avoid negative outcomes. There is need for comprehensive, educational health programs, aimed at reducing BV prevalence and guide the planning and resource allocation of decision makers for future interventions and research.

## Abbreviations

ANC: Antenatal care
BV: Bacterial vaginosis
CMA: Centre Medicaled’Arrondissement; Sub-divisional Medica Center
HVS: High vaginal swab
KHD: Kumba Health District
SED: Sexually Enhanced Disease
STD: Sexually Transmitted Disease

## References

[1] Bertini M. Bacterial Vaginosis and Sexually Transmitted Diseases: Relationship and Management. IntechOpen. 2017. 10.5772/intechopen.69258.

[2] Kenyon C, Colebunders R, Crucitti T (2013). The global epidemiology of bacterial vaginosis: a systematic review. Am J Obstet Gynecol.

[3] Alcendor DJ (2016). Evaluation of health disparity in bacterial vaginosis and the implications for HIV-1 Acquisition in African American Women. Am J Reprod Immunol.

[4] Machado D, Castro J, Martinez-de-Oliveira J, Nogueira-Silva C, Cerca N (2017). Prevalence of bacterial vaginosis in Portuguese pregnant women and vaginal colonization by *Gardnerella vaginalis*. PeerJ.

[5] Hyman RW, Fukushima M, Diamond L, Kumm J, Giudice LC, Davis RW (2005). Microbes on the human vaginal epithelium. PNAS..

[6] Achondou AE, Fumoloh FF, Aseneck AC, Awah AR, Utokoro AM (2016). Prevalence of bacterial vaginosis among sexually active women attending the CDC central clinic Tiko, south west region, Cameroon. Afr J Infect Dis.

[7] Bitew A, Abebaw Y, Bekele D, Mihret A. Prevalence of bacterial vaginosis and associated risk factors among women complaining of genital tract infection. Int J Microbiol 2017; 2017: 4919404. 10.1155/2017/4919404 PMC5558670 28831285

[8] Afolabi BB, Olusanjo EM, Oyinlola OO (2016). Bacterial vaginosis and pregnancy outcome in Lagos, Nigeria. Open Forum Infect Dis.

[9] Li XD, Tong F, Zhang XJ, Pan WJ, Chen ML, Wang CC, Li X, Gao GP, Sun L, Sun YH (2015). Incidence and risk factors of bacterial vaginosis among pregnant women: a prospective study in Maanshan city, Anhui Province, China. J Obstet Gynaecol Res.

[10] Desseauve D, Chantrel J, Fruchart A, Khoshnood B, Brabant G, Ancel PY, Subtil D (2012). Prevalence and risk factors of bacterial vaginosis during the first trimester of pregnancy in a large French population-based study. Eur J Obstet Gynecol Reprod Biol.

[11] Shayo PA, Kihunrwa A, Massinde AN, Mirambo A, Rumanyika R, Ngwalida N, Gumodoka B, Kidola J, Magoma M (2012). Prevalence of bacterial vaginosis and associated factors among pregnant women attending at Bugando medical Centre, Mwanza, Tanzania. Tanzania J Health Res.

[12] Verstraelen H, Verhelst R, Vaneechoutte M, Temmerman M (2010). The epidemiology of bacterial vaginosis in relation to sexual behaviour. BMC Infect Dis.

[13] Fethers KA, Fairley CK, Hocking JS, Gurrin LC, Bradshaw CS (2008). Sexual risk factors and bacterial vaginosis: a systematic review and meta-analysis. Clin Infect Dis.

[14] Mengistie Z, Woldeamanuel Y, Asrat D, Adera A (2014). Prevalence of bacterial vaginosis among pregnant women attending antenatal care in Tikur Anbessa University hospital, Addis Ababa, Ethiopia. BMC Res Notes.

[15] Yzeiraj-Kalemaj L, Shpata V, Vyshka G, Manaj A. Bacterial vaginosis, educational level of pregnant women, and preterm birth: a case-control study. ISRN Infect Dis 2013; 2013, Article ID 980537.

[16] Das TR, Fatema K, Chowdhury S, Noor F, Ara R, Chakma B, Parveen M, Ali MJB (2017). Association of Bacterial Vaginosis with preterm delivery. J Bangladesh Col Physic Surg.

[17] Kiran CK, Kandati J, Ponugoti M (2017). Prevalence of bacterial vaginosis in preterm and termlabour: a one year study. Int J Reprod Contracept Obstet Gynecol.

[18] Işik G, Demirezen S, Dönmez GH, Beksaç SM (2016). Bacterial vaginosis in association with spontaneous abortion and recurrent pregnancy losses. J Cytol.

[19] Haggerty CL, Hillier SL, Bass DC, Ness RB (2004). Bacterial vaginosis and anaerobic bacteria are associated with endometritis. Clin Infect Dis.

[20] Mullick S, Watson-Jones D, Beksinska M, Mabey D (2005). Sexually transmitted infections in pregnancy: prevalence, impact on pregnancy outcomes, and approach to treatment in developing countries. Sex Transm Infect.

[21] Forcey DS, Vodstrcil LA, Hocking JS, Fairley CK, Law M, McNair RP, Bradshaw CS (2015). Factors associated with bacterial vaginosis among women who have sex with women: a systematic review. PLoS One.

[22] Bautista CT, Wurapa E, Sateren WB, Morris S, Hollingsworth B, Sanchez JL (2016). Bacterial vaginosis: a synthesis of the literature on etiology, prevalence, risk factors, and relationship with chlamydia and gonorrhea infections. Mil Med Res.

[23] Atashili J, Poole C, Ndumbe PM, Adimora AA, Smith JS (2008). Bacterial vaginosis and HIV acquisition: a meta-analysis of published studies. AIDS..

[24] Mirmonsef P, Krass L, Landay A, Spea GT (2012). The role of bacterial vaginosis and *Trichomonas* in HIV transmission across the female genital tract. Curr HIV Res.

[25] Nsagha DS, Zofou D, Assob JCN, Njunda AL, Nchang CD, MvoNgum N, Weledji EP, NgoweNgowe M (2015). The epidemiology of *Trichomonas vaginalis, Gardnerella vaginalis* and *Candida albicans* co-infections in women attending the Yaounde University teaching hospital. Am J Epidemiol Infect Dis.

[26] Kesah FNC, Payne VK, Asakizi A (2013). Prevalence and etiology of sexually transmitted infections in a gynecologic unit of a developing country. Ann Trop Med Pub Health.

[27] Mbu ER, Kongnyuy EJ, Mbopi-Keou FX, Tonye RN, Nana PN, Leke RJI (2008). Gynaecological morbidity among HIV positive pregnant women in Cameroon. Reprod Health.

[28] Daniel WW (1999). Biostatistics: A Foundation for analysis in the health sciences.

[29] Nugent RP, Krohn MA, Hillier SL (1991). Reliability of diagnosing bacterial vaginosis is improved by standardized method of gram stain interpretation. J Clin Microbiol.

[30] Amsel R, Totten PA, Spiegel CA, Chen KC, Eschenbach D, Holmes KK (1983). Non-specific vaginitis: diagnostic criteria and microbial and epidemiologic associations. AJM..

[31] Nergis TAJ, Alam MSU, Sarah, Bajwa Z, Waheed A, Ullah E (2014). Bacterial vaginosis in pregnant women and its diagnosis using Amsel’s Clinical Criteria and Nugent’s method. P J M H S.

[32] Redelinghuys MJ, Ehlers MM, Dreyer AW, Lombaard H, Kock MM. Prevalence of genital mycoplasmas and bacterial vaginosis in pregnant women in Gauteng, South Africa 2013; 10.1136/sextrans-2013-051184.0495.

[33] Abdelaziz ZA, Ibrahim ME, Bilal NE, Hamid ME (2014). Vaginal infections among pregnant women at Omdurma maternity Hospital in Khartoum, Sudan. J Infect Dev Ctries.

[34] Awoniyi AO, Komolafe OI, Bifarin O, Olaniran O (2015). Bacterial vaginosis among pregnant women attending a primary health care center in lle-Ife, Nigeria. Glo Adv Res J Med Med Sci.

[35] Ajani G, Oduyebo O, Haruna M, Elikw C (2012). Nugent scores for pregnant women in a tertiary institution in Nigeria. AdvMicrobiol..

[36] Ranjit E, Raghubanshi BR, Maskey S, Parajuli P. Prevalence of bacterial vaginosis and its association with risk factors among nonpregnant women: a hospital based study. Int J Microbiol. 2018; Article ID 8349601 10.1155/2018/8349601. 10.1155/2018/8349601 PMC5859802 29692813

[37] Sobel JD, Subramanian C, Foxman B, Fairfax M, Gygax SE (2013). Mixed vaginitis-more than coinfection and with therapeutic implications. Curr Infect Dis Rep.

[38] Ibrahim SM, Bukar M, Galadima GB, Audu BM, Ibrahim HA (2014). Prevalence of bacterial vaginosis in pregnant women in Maiduguri, North-Eastern Nigeria. Niger J Clin Pract.

[39] Nwadioha SI, Egah DZ, Banwat EB, Aloa OO (2010). Microbial agents of abnormal vaginal discharge in mothers attending PHC centres of Jos, Nigeria. J Clin Med Res.

[40] Mitchell C, Moreira C, Fredricks D, Paul K, Caliendo AM, Kurpewski J, Ingersoll J, Cu-Uvin S. Detection of fastidious vaginal bacteria in women with HIV infection and bacterial vaginosis. Infect Dis Obstet Gyneco. 2009;236919. 10.1155/2009/236919 PMC2777244 19920869

[41] Schellenberg JJ, Card CM, Ball TB, Mungai JN, Irungu E, Kimani J, Jaoko W, Wachihi C, Fowkes KR, Plummer FA (2012). Bacterial vaginosis, HIV serostatus and T-cell subset distribution in a cohort of East African commercial sex workers: retrospective analysis. AIDS.

[42] Amin AA. Rural Poverty and Agricultural Development in Cameroon. Paper presented at a Colloqium organized by Unesco, Universite Montesquieu-Bordeau IV – Unesco Paris. 22 & 23 November 2001.

